# Early detection of deterioration in COVID-19 patients by continuous ward respiratory rate monitoring: a pilot prospective cohort study

**DOI:** 10.3389/fmed.2023.1243050

**Published:** 2023-10-31

**Authors:** Eva Rivas, Manuel López-Baamonde, Josep Sanahuja, Elena Del Rio, Tomeu Ramis, Anna Recasens, Antonio López, Marilyn Arias, Stylianos Kampakis, Timo Lauteslager, Osama Awara, Edward J. Mascha, Alex Soriano, Joan Ramon Badía, Pedro Castro, Daniel I. Sessler

**Affiliations:** ^1^Department of Anesthesia, Hospital Clinic of Barcelona, IDIBAPS, Universidad de Barcelona, Barcelona, Spain; ^2^Department of Outcomes Research, Cleveland Clinic, Cleveland, OH, United States; ^3^Department of Anesthesiology and Critical Care, Hosptial Universitary Son Espases, Mallorca, Spain; ^4^Department of Anesthesiology, Hospital del Mar. Institut Hospital del Mar d’Investigacions Mèdiques (IMIM), Universitat Autònoma de Barcelona, Barcelona, Spain; ^5^Circadia Technologies, Ltd., London, United Kingdom; ^6^Department of Quantitative Health Sciences, Cleveland Clinic, Cleveland, OH, United States; ^7^Department of Infectious Disease, Hospital Clinic of Barcelona, IDIBAPS, Universidad de Barcelona, CIBERINF, Barcelona, Spain; ^8^Department of Pneumology, Hospital Clinic of Barcelona, IDIBAPS, Universidad de Barcelona, Barcelona, Spain; ^9^Medical Intensive Care Unit, Hospital Clinic of Barcelona, IDIBAPS, Universidad de Barcelona, Barcelona, Spain

**Keywords:** respiratory rate, continuous monitoring, respiratory failure, COVID-19, hospitalization ward

## Abstract

**Background:**

Tachypnea is among the earliest signs of pulmonary decompensation. Contactless continuous respiratory rate monitoring might be useful in isolated COVID-19 patients admitted in wards. We therefore aimed to determine whether continuous monitoring of respiratory patterns in hospitalized patients with COVID-19 predicts subsequent need for increased respiratory support.

**Methods:**

Single-center pilot prospective cohort study in COVID-19 patients who were cared for in routine wards. COVID-19 patients who had at least one escalation of pulmonary management were matched to three non-escalated patients. Contactless respiratory monitoring was instituted after patients enrolled, and continued for 15 days unless hospital discharge, initiation of invasive mechanical ventilation, or death occurred. Clinicians were blinded to respiratory rate data from the continuous monitor. The exposures were respiratory features over rolling periods of 30 min, 24 h, and 72 h before respiratory care escalation. The primary outcome was a subsequent escalation in ventilatory support beyond a Venturi mask.

**Results:**

Among 125 included patients, 13 exhibited at least one escalation and were each matched to three non-escalated patients. A total of 28 escalation events were matched to 84 non-escalation episodes. The 30-min mean respiratory rate in escalated patients was 23 breaths per minute (bpm) ranging from 13 to 40 bpm, similar to the 22 bpm in non-escalated patients, although with less variability (range 14 to 31 bpm). However, higher respiratory rate variability, especially skewness over 1 day, was associated with higher incidence of escalation events. Our overall model, based on continuous data, had a moderate accuracy with an AUC 0.81 (95%CI: 0.73, 0.88) and a good specificity 0.93 (95%CI: 0.87, 0.99).

**Conclusion:**

Our pilot observational study suggests that respiratory rate variability as detected with continuous monitoring is associated with subsequent care escalation during the following 24 h. Continuous respiratory monitoring thus appears to be a valuable increment over intermittent monitoring.

**Strengths and limitations:**

Our study was the initial evaluation of Circadia contactless respiratory monitoring in COVID-19 patients who are at special risk of pulmonary deterioration. The major limitation is that the analysis was largely *post hoc* and thus needs to be confirmed in an out-of-sample population.

## Background

The COVID-19 pandemic has affected almost 600 million people and is on track to kill 6.5 million people worldwide (1). During the first wave, most patients experienced mild–moderate symptoms, but up to 10% required hospitalization (2, 3). Among those admitted to the hospital, depending on various comorbidities, a substantial fraction progressed to respiratory failure requiring ventilatory support (4).

Early detection of deteriorating patients may improve management and outcomes. Tachypnea is among the earliest signs of pulmonary decompensation. For example, respiratory rates exceeding 35 breaths/min predict life-threatening events within 8 h (odds ratio [OR], 31; 95%CI: 8, 130) (5). And in COVID-19 patients, tachypnea has been associated with progression to mechanical ventilation (6), ICU admission (7), and mortality (2).

Respiratory depression (8) and hypoxemia (9) are common among hospitalized patients, even those without COVID-19. Furthermore, nurses assessing vital signs at 4-h intervals in hospital wards miss half of all serious and prolonged hypotensive episodes (10), and more than 90% of serious and prolonged desaturation episodes (9, 10). An additional consideration is that healthcare professionals’ manually obtained respiratory rate measurements are often inaccurate (11, 12), and recorded values often default to either 18 or 20 breaths per minute (bpm) (13). Automated continuous monitoring tools, although not immune to artifacts, have well-established accuracy and artifact-caused errors can be averaged out through continuous monitoring.

Recent advances in sensor technologies have made it possible to obtain respiratory rate without direct attachment to patients or their beds. Such contactless monitoring technologies, by virtue of not requiring effort from patient or healthcare professionals, facilitate continuous vital sign monitoring (14, 15). Use of continuous respiratory rate data has two additional advantages over infrequent spot-check data for prediction of clinical events. Firstly, naturally occurring short term variation in breathing rate is fully sampled and can thus be averaged out, resulting in a more precise estimate of mean respiratory rate (15). Secondly, variation of respiratory rate over time may contain clinically relevant information. For example, trends and variations in respiratory rate across various intervals predict adverse events (16–18). The Circadia Contactless Breathing Monitor (model C100, Circadia Technologies, Ltd., London, United Kingdom) uses radar to estimate respiratory rate in awake and sleeping patients. A feature of the monitor is that it is contactless, thus facilitating long-term monitoring in hospital wards or at home without the discomfort associated with wearable devices or the need to replace batteries (19).

Undetected vital sign abnormalities may be especially likely in COVID-19 wards because patient isolation and the need of personal protection equipment preclude facile clinical assessments. Automated and continuous monitoring of respiratory function may thus be especially helpful in COVID-19 patients sick enough to require hospitalization (20). We therefore conducted a single-center pilot prospective cohort study in hospitalized COVID-19 patients. Our primary goal was to assess the extent to which continuous untethered ward respiratory rate patterns identify patients who require escalation of pulmonary management 24 h ahead of time, defined by progression to high-flow oxygen, non-invasive mechanical ventilation, invasive mechanical ventilation, or death. Specifically, we sought to evaluate whether identifiable respiratory rate patterns are meaningfully associated with ventilatory decompensation in COVID-19 patients.

## Methods

Our prospective observational pilot study was conducted with approval from the Hospital Clinic of Barcelona IRB (Ethics Committee’s approval/ID: HCB 2020/0666). Written informed consent was obtained from participating patients. The study was performed in accordance with relevant guidelines and regulations.

### Subject selection

We enrolled adults of any race and ethnicity who were admitted to a routine nursing ward with a diagnosis of SARS-CoV-2 infection, confirmed by a reverse-transcription polymerase chain reaction test. We excluded patients younger than 18 years old and pregnant women. Since we targeted patients at risk of clinical deterioration who were eligible for non-invasive and invasive mechanical ventilation support, we also excluded patients in whom escalation to life-sustaining respiratory support include non-invasive modalities such as high-flow nasal cannula oxygen therapy (HFNCO) but stop short before intubation and invasive mechanical ventilation because treatment was unlikely to prove helpful.

### Protocol

We included patients who had COVID-19 and were hypoxemic, defined as arterial pressure of oxygen (PaO_2_) <80 mmHg or pulse oximeter saturation (SpO_2_) <90% while breathing ambient air. All were hospitalized for treatment and required supplemental oxygen, to ensure a SpO_2_ over 91%.

According to our hospital guidelines for respiratory failure in COVID-19 patients, patients were considered to be in respiratory failure and candidates for escalation of pulmonary care when they developed moderate-to-severe shortness-of-breath with use of accessory muscles or paradoxical abdominal movements and tachypnea of ≥30 bpm; when the PaO_2_/Inspired fraction of oxygen (FiO_2_) ratio was ≤200; if a FiO_2_ > 0.4 was needed to maintain a SpO_2_ > 92%; or when pH was <7.35 accompanied by a PaCO_2_ exceeding 45 mmHg.

HFNCO was generally the initial treatment, followed by Non-Invasive Mechanical Ventilation (NIMV). Patients were escalated to invasive mechanical ventilation if they did not improve and had a ROX index [(SpO_2_/FiO_2_) / respiratory rate] <3 within 2 h, <3.5 within 6 h, or < 4 within 12 h after HFNCO and NIVM initiation (21).

### Measurements

Demographic, morphometric, and clinical characteristics, as well as nurse-recorded vital signs including blood pressure, heart rate, respiratory rate, oxygen saturation, ventilation mode, supplemental oxygen concentration, and delivery system were recorded.

Pulmonary management was recorded, including use of HFNCO, NIMV, invasive mechanical ventilation, and clinically detected deterioration. We also recorded episodes when nurses requested non-routine physician or ICU team evaluation, transfer to a higher-acuity unit, and discharge disposition including vital status. Arterial blood gas analysis was not mandated per protocol, but available values were extracted from medical records.

Continuous respiratory monitoring was performed using the Circadia Contactless Breathing Monitor (model C100, Circadia Technologies, Ltd., London, United Kingdom). The Circadia Monitor is a radar-based device, and relies on tracking of time-of-flight variations of reflected radar pulses to estimate the rate of chest wall motion. The Circadia C100 System (comprising the Monitor and cloud services) is FDA-cleared as a continuous breathing frequency monitor intended for the professional healthcare facility environment. Accuracy of the system (specified as ±2 breaths per minute) has been validated by direct comparison against manually scored end-tidal CO_2_ and ventilatory effort reference data, both during spot measurements and continuous monitoring in awake and sleeping patients (19). Respiratory rate is computed at 3-s intervals using proprietary signal processing algorithms on the Monitor, and wirelessly transmitted to the cloud. Algorithms are designed to automatically reject inaccurate or erroneous respiratory rate values, which makes the system suitable for continuous and unsupervised use. Using digital time-gating methods, it is ensured that only signals corresponding to the patient’s bed are being recorded by the device. Furthermore, periodicity and waveform of recorded signals are analyzed in real-time to determine whether the signal corresponds to a breathing pattern. Practically, this means that the Monitor will only output respiratory rate during low-motion conditions while the patient is in bed, and suppresses outputs while respiratory rate cannot reliably be obtained (e.g., during patient absence, movement or noise sources). Respiratory rate as outputted by the Monitor can thus directly be used for subsequent feature extraction and modeling steps. For the current study however, the system was modified such that raw sensor data were recorded and transmitted to cloud-based storage, for offline processing. Production-equivalent signal processing algorithms were applied to raw sensor data to compute respiratory rate, with the only exception that the usual detectable range was increased from the FDA-cleared 7–38 breaths per minute to 5–70 breaths per minute. This broader range was found to be more suitable considering the higher acuity of this clinical environment, and allowed to capture the full range of respiratory patterns in study participants. We began Circadia monitoring as soon as practical after patients consented to participate. Circadia Monitors were positioned about 1.5 meters from patients. Monitoring continued for 15 days, unless aborted for hospital discharge, initiation of invasive mechanical ventilation, or death. Respiratory rate data from the Circadia Monitor was not available to clinicians who managed participating patients.

### Exposure and outcomes

The primary outcome was pulmonary decompensation, defined as an escalation in the pulmonary care beyond Venturi-mask support. The following levels of respiratory support were defined, from less to more severe: (1) ambient air; (2) nasal cannula; (3) Venti-mask; (4) HFNCO; (5) NIMV; (6) invasive mechanical ventilation; and (7) death. Our primary aim was to assess whether the information extracted from respiratory rate continuous monitoring is associated with respiratory care escalation 24 h in advance in hospitalized COVID-19 patients.

The exposure was short-term and long-term respiratory patterns associated with respiratory care escalation. Respiratory patterns (variability and fluctuations over time) were captured through features, which included respiratory rate mean, respiratory rate standard deviation, kurtosis of the respiratory rate, skewness of the respiratory rate, and trend (extracted from the slope of a linear regression model), all computed over three rolling periods, 30 min, 24 h and 72 h in duration, occurring 24 h prior to respiratory care escalation (Supplementary Table 1).

### Data analysis

Analysis was restricted to subjects who had successful Circadia monitoring for at least half of their hospitalization. We considered patients to have escalated respiratory care when they required an increment in their ventilatory support category, with others falling into the non-escalating category. Patients could experience multiple escalation episodes within a day. However, we considered only the initial escalation within each 48-h period to avoid situations where a prior de-escalation of treatment was quickly unsuccessful, rather than representing a true new worsening of the underlying pulmonary function. We also excluded patients who de-escalated ventilatory support after joining the study. We considered a patient to be de-escalating if they required HFNCO or NIMV at study inclusion and thereafter progressively improved (Supplementary Figure 1).

Patients who required ventilatory support escalation were matched to three non-escalating patients on baseline variables (Table 1) using minimum Euclidean distance. Escalation episodes in each patient were matched to a similar point in the three matched non-escalation patients based on time since admission. When the exact corresponding time was unavailable because the non-escalation patient was already discharged at this time point, the last available time window was used. A non-escalated patient could be matched with more than 1 escalated patient (Supplementary Table 2).

**Table 1:** Patient demographic, morphometric, and clinical characteristics.

	Before matching			After matching		
	Escalation			Escalation		
	YES (*N* = 13)	NO (*N* = 112)	*p* value	YES (*N* = 13)	NO (*N* = 26)	*p* value
Age, years	67 ± 17	61 ± 14	0.16	67 ± 17	63 ± 12	0.40
Female, *n* (%)	10 (77)	73 (65)	0.54	10 (77)	20 (77)	1
BMI, kg/m^2^	31 ± 5	28 ± 5	0.043	31 ± 5	29 ± 4	0.18
Medical history						
Arterial hypertension, *n* (%)	8 (62)	56 (50)	0.43	8 (62)	19 (73)	0.49
Diabetes mellitus, n (%)	3 (23)	18 (16)	0.46	3 (23)	6 (23)	1
Ischemic cardiac disease, *n* (%)	1 (8)	10 (9)	1	1 (8)	2 (8)	1
Chronic heart failure, *n* (%)	2 (15)	2 (2)	0.053	2 (15)	1 (4)	0.25
COPD, *n* (%)	2 (15)	11 (10)	0.62	2 (15)	3 (12)	1
Asthma, *n* (%)	1 (8)	10 (9)	1	1 (8)	1(4)	1
Neurological disease, *n* (%)	1 (8)	12 (11)	1	1 (8)	1(4)	1

Variables were summarized as means ± SDs and N(%), as appropriate. BMI, body mass index; COPD, Chronic Obstructive Pulmonary Disease. Two-sample *t*-test, chi-square and Fisher’s exact test were used for comparisons, as appropriate. *p* < 0.05 was considered significant.

Respiratory rate was sampled by the Circadia Monitor at 3-s intervals. This permitted capture of not only day-to-day changes in absolute levels of respiratory rate, but also within-day variation of respiratory rate (often missed by spot measurements). Examples of within-day variation are diurnal and ultradian rhythms (related to rest-activity patterns and the circadian rhythm) and acute or short-term changes in respiratory rate (related to patient activity, treatment, decompensation, or deterioration). To express both short-term and long-term patterns in respiratory rate mathematically, a set of features was derived from continuous respiratory rate data. Features were computed using a 30-min rolling analysis window, sampled at 30-min intervals, through various aggregation methods including the mean. The mean respiratory rate over 30 min was further aggregated using additional rolling windows of 24 and 72 h, also sampled at 30-min increments, to obtain the 24-h and 72-h features. Features included the mean, minimum, maximum, and the trend (e.g., level of increase or decrease in respiratory rate), within the feature window. Additional features described the distribution of respiratory data within the analysis window: standard deviation (expressing the level of variance, or ‘width’ of the distribution), skewness (a measure of asymmetry of the respiratory rate distribution), and kurtosis (describing how heavy the tails of the distribution are, and thus how many respiratory rate outliers are observed). Although the latter statistical features may not invoke clinical intuition, they were found to be highly suitable to describe how erratic and volatile continuous respiratory rate data was in the rolling analysis window.

Since some patients had multiple escalation events, we estimated the standard error and confidence intervals of the associations of interest using bootstrap resampling of the logistic regression model (10,000 runs, sampling episode ID with replacement and using the percentile method), while at the same time adjusting for confounding variables. We also used the variance inflation factor (VIF) to estimate collinearity between respiratory features. Higher values of VIF (above 10) indicate the existence of multicollinearity, meaning that such variables are highly correlated and should not simultaneously be included in the model (21). The model was fitted using the *glm* package in R (version 3.6.0).

### Statistical analysis

We used a multivariable logistic regression model to assess the degree to which various respiratory rate features were associated with respiratory care escalation occurring 24 h later. The criterion for a predictor to be significant was a value of *p* < 0.05 in the multivariable logistic regression model, uncorrected for multiple comparisons. Performance of the model was measured using traditional measures of diagnostic accuracy, specifically c-statistic from the logistic regression model [area under the receiver operating characteristic curve (ROC-AUC)], sensitivity, specificity, positive predictive value (PPV), and negative predictive value (NPV). We assumed a decision threshold >0.5 from the logistic regression model predicted escalation.

### Sample size considerations

There was no *a priori* sample size estimate for our pilot study. Instead, we enrolled all qualifying and consenting patients over the study period. The achieved sample size of 125 patients, each with repeated measurements, allowed us to form confidence intervals for the parameters of interest with sufficient precision to evaluate early prediction of respiratory escalation. The width of the diagnostic accuracy confidence intervals range from 0.12 to 0.32, which we deem sufficiently narrow to make meaningful conclusions. We do not conduct a *post-hoc* power analysis *per se* because our main goal is estimation of the diagnostic accuracy parameters as opposed to testing.

## Results

**Figure 1:**
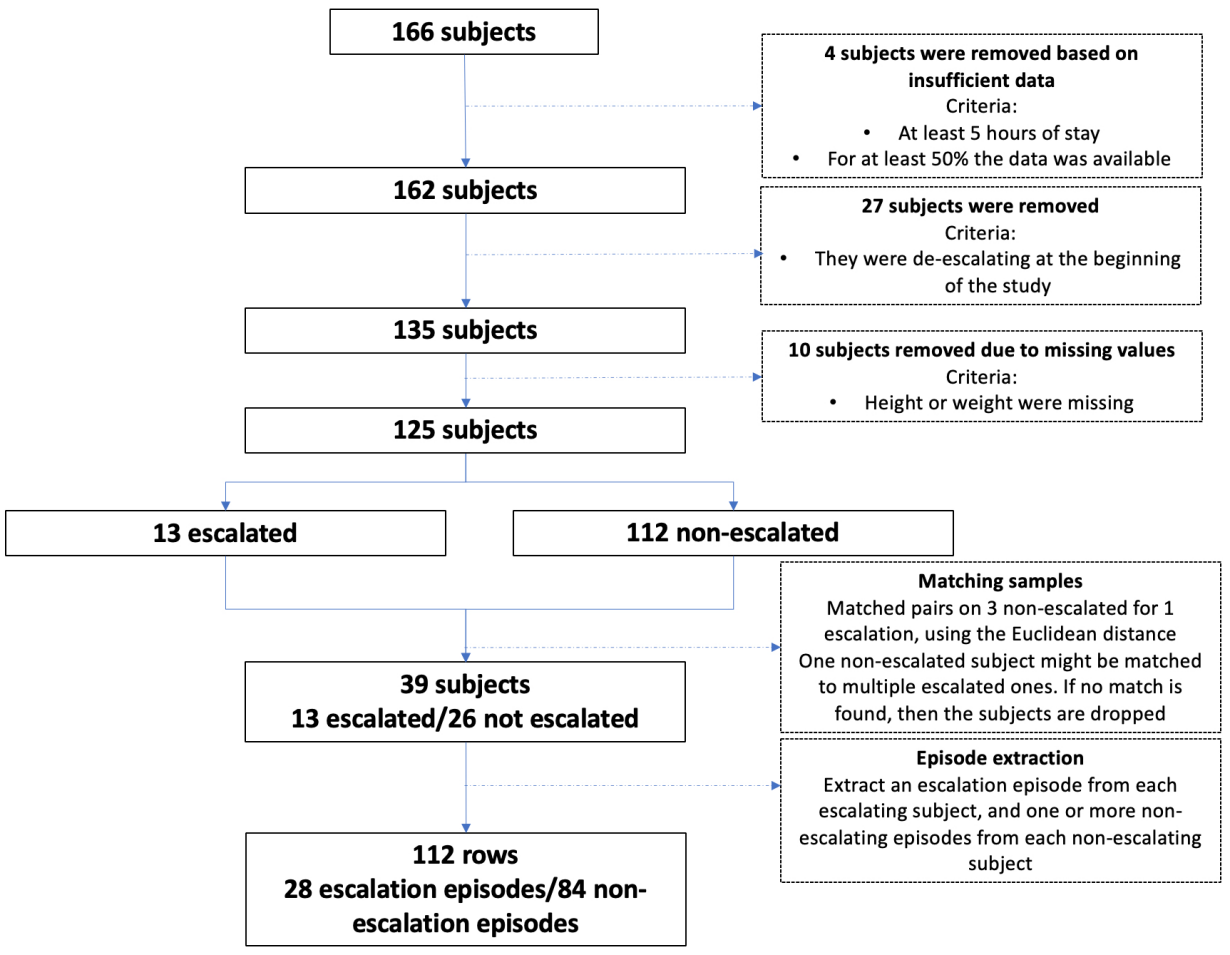
Subject selection flow chart.

Between January 15th, 2021 and May 29th, 2021, we enrolled 166 patients. We excluded 14 patients due to data quality issues (10 because of missing demographic characteristics and 4 because of Circadia recordings failures). We also excluded 27 subjects who were in the process of de-escalating ventilatory support when they joined the study. We finally included 125 subjects in the study (Figure 1).

Thirteen patients exhibited at least one escalation, and each was matched to 3 non-escalated patients. Six of the patients who required care escalations experienced 2–5 escalation episodes. There was thus a total of 28 escalation episodes that were matched to 84 non-escalation episodes (Supplementary Table 2). Demographic and clinical characteristics before and after patients matching are shown in Table 1.

The 30 min window analysis showed that the mean respiratory rate 24 h prior to an escalation episode was 23 bpm, with a wide range from 7 to 40 bpm, while in the non-escalation patients the mean respiratory rate was 22 bpm but the range was narrower, from 14 to 31 bpm. Escalated patients showed significantly more variability in the respiratory rate than non-escalated patients with higher respiratory rate standard deviation and skewness (Table 2).

**Table 2:** Respiratory rate features in escalated and non-escalated patients.

	Escalated (28 events)			Non-escalation (84 events)			Mean difference (95% CI)	*p* value
Mean, 30 min	22.5	±	1.2	22.0	±	0.5	−0.50 (−3.10, 2.11)	0.702
SD, 30 min	2.1	±	0.2	1.6	±	0.1	−0.57 (−1.04, −07)	**0.022**
Skew, 30 min	0.5	±	0.2	−0.1	±	0.1	−0.54 (−0.98, −0.086)	**0.020**
Kurtosis, 30 min	1.0	±	0.5	0.8	±	0.3	−0.27 (−1.60, 1.06)	0.689
Mean, 1 day	23.0	±	0.1	21.9	±	0.4	−1.16 (−3.31, 0.99)	0.280
SD, 1 day	2.9	±	0.3	2.6	±	0.1	−0.26 (−0.80, 0.28)	0.341
Skew, 1 day	0.4	±	0.1	0.1	±	0.1	−0.31 (−0.54, −0.08)	**0.010**
Kurtosis, 1 day	0.0	±	0.3	−0.3	±	0.1	−0.33 (−0.84, 0,18)	0.202
Trend, 1 day	−0.7	±	2.2	−1.9	±	0.5	−1.18 (−5.74, 3.38)	0.600
Mean, 3 days	23.6	±	1.0	22.0	±	0.4	−1.52 (−3.48, 0.45)	0.127
SD, 3 days	3.4	±	0.3	2.7	±	0.1	−0.62 (−1.17, −0.07)	**0.027**
Skew, 3 days	0.3	±	0.1	0.1	±	0.5	−0.21 (−0.41, −0.01)	**0.037**
Kurtosis, 3 days	−0.1	±	0.2	−0.2	±	0.1	−0.11 (−0.44, 0.23)	0.526
Trend, 3 days	−1.5	±	0.9	−0.6	±	0.2	0.89 (−1.09, 2.88)	0.364

Data expressed as means ± standard errors. All units are in breaths per minute, except for Trend (breaths per minute per day). T-tests were used for comparisons. *p* < 0.05 (in bold) was considered a significant difference.

**Figure 2:**
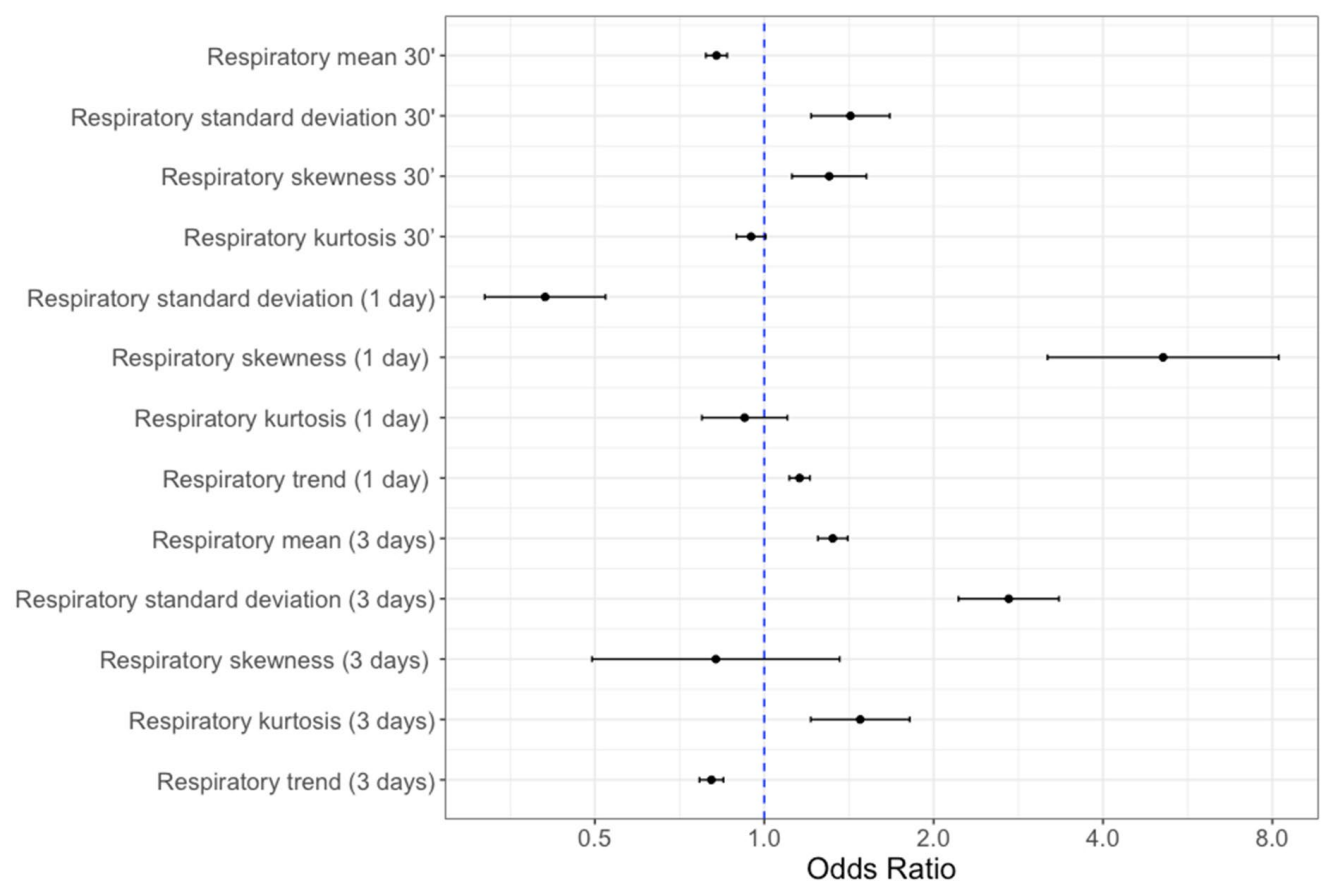
Forest plot of respiratory rate features odds ratio (95% confidence intervals) of being associated with an escalation event within 24 h thereafter. Higher values indicate higher odds of escalation. All units are in breaths per minute, except respiratory trend (breaths per minute per day).

The variance inflation factor (VIF) for the mean of the respiratory rate over 1 day was 20.4, while all inflation factors were < 5. Therefore, mean respiratory rate over 1 day was removed, and our final model included 13 respiratory features. The probability that the respiratory rate features were associated with an escalation event 24 h later is shown in Figure 2. When assessing the 30 min window, no significant association was found between increased mean respiratory rate and risk of escalation, despite higher respiratory rate found prior to escalation events. The most remarkable result was that features describing continuous respiratory rate distribution (in particular standard deviation and skewness) were most strongly associated with higher incidence of an escalation event (Figure 2).

**Figure 3:**
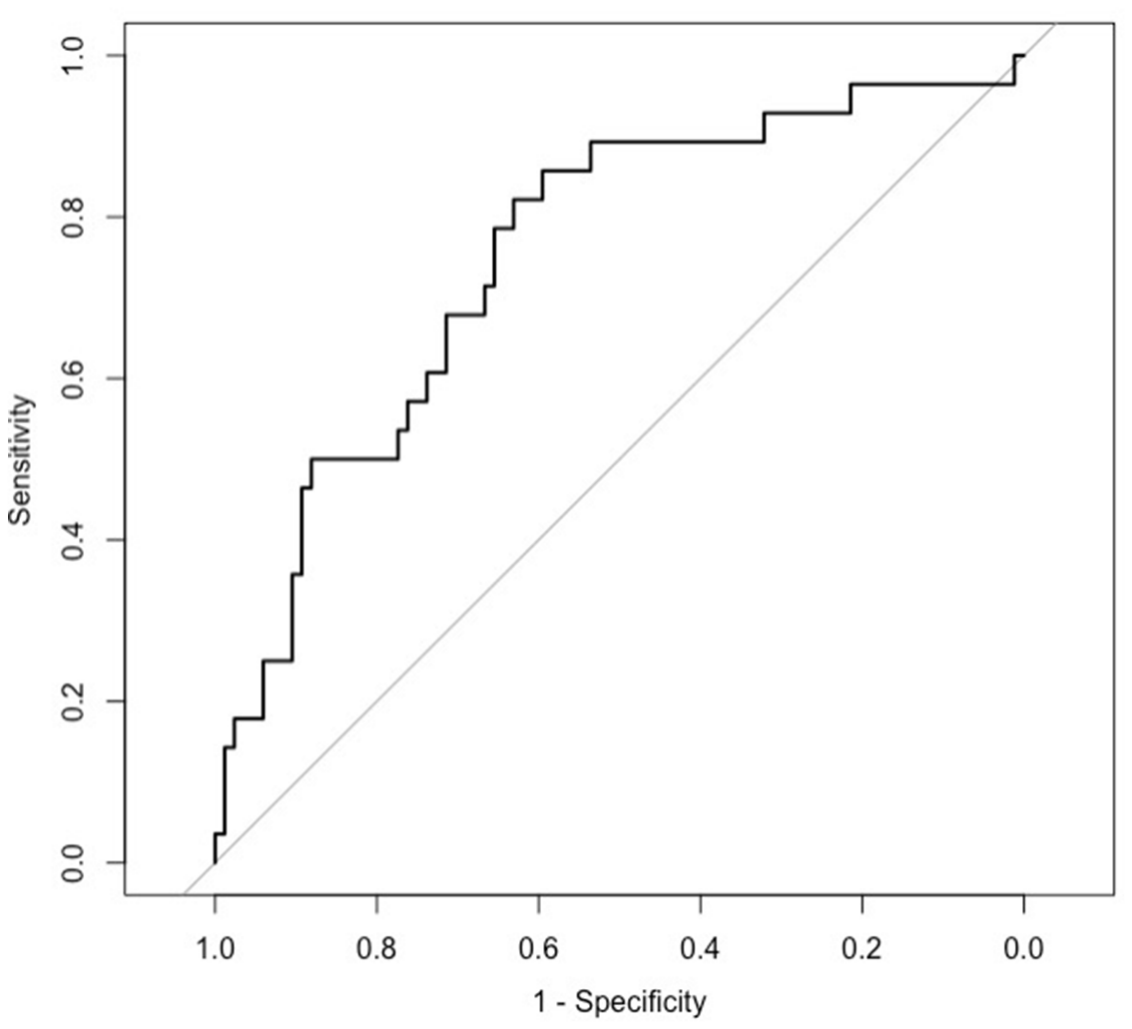
Receiver operating characteristics curve for full model.

Our overall model had a moderate accuracy with an AUC 0.81 (95% CI: 0.73, 0.88) and a good specificity 0.93 (95% CI: 0.87, 0.99) being able to correctly detect 93% of patients who did not escalate (Table 3; Figure 3).

**Table 3:** Performance of the full logistic regression model assessing the association between all respiratory features and subsequent respiratory care escalation within 24 h.

Metric	Estimate (95% CI)
AUC	0.81 (0.73, 0.88)
Sensitivity	0.42 (0.26, 0.57)
Specificity	0.93(0.87, 0.99)
PPV	0.85 (0.67, 0.99)
NPV	0.62(0.52, 0.72)

AUC, Area Under the Curve; PPV, Positive predictive value; NPV, Negative predictive value. All metrics were calculated after a complete fit of the model. The decision threshold was 0.5.

## Discussion

Many COVID-19 patients are unstable, and respiratory status often worsens over time. For example, 13 of 125 (10%) of our ward patients required 28 meaningful enhancements in ventilatory support such as transition to HFNCO or ICU admission. The clinical challenge, of course, is to identify patients at risk as early as practical so that resources and attention can be wisely allocated.

Early detection of respiratory failure and prompt initiation of ventilatory support reduces COVID-19 complications and mortality (2). Tachypnea predicts the need of mechanical ventilation (6) and ICU admission (7) in COVID-19 patients. Moreover, tachypnea with more than 24 breaths per minute is associated with a 5-fold increase in mortality (2). Accurate and frequent assessments of respiratory rate are thus an important part of COVID-19 care (22), and presumably also are for other serious progressive respiratory infections.

Vital signs are normally assessed at 4-8-h intervals on hospital wards, a frequency that has not much increased in the last half-century, although hospitalized patients are now frequently far sicker than previously. Because of their special ventilatory risk, COVID-19 patients seem likely to benefit from more frequent monitoring but may actually get less because isolation precludes easy access. The Circadia Contactless Breathing Monitor we used is an alternative approach that provides a continuous estimate of respiratory rate.

An advantage of continuous monitoring is that in addition to respiratory rate *per se*, we were able to simultaneously evaluate various measures of respiratory rate variation over time. Features describing the distribution of consecutive respiratory rate measurements (skew and standard deviation in particular) were most strongly associated with escalation episodes. Analysis of these features requires continuous monitoring as they cannot be estimated from intermittent spot measurements alone. Our results therefore suggest that continuous monitoring may allow for improved detection of ventilatory deterioration.

A feature of our results is that while respiratory rate *per se* was not especially predictive of future respiratory decline, the standard deviation and skewness of the respiratory rate were. Changes in variability similarly indicates abnormal conditions in other circumstances. For example, heart-rate variability is a well-established indicator of autonomic stress, whereas heart rate alone is less useful (23). Similarly, heart-rate variability predicts atrial fibrillation (24, 25) and is used as a measure of nociception (25). One potential explanation for these findings is that variability in respiratory rate may represent physiological decompensation, whereas mean respiratory rate changes are blunted through escalation of respiratory support.

Our model, based on 24-h pre-escalation periods, had an area under the receiver operating characteristics curve of 0.81 which indicates moderate predictive accuracy and potential clinical utility. The high specificity allows clinicians the possibility of accurately identify patients at low risk of respiratory de-compensation and thus focus efforts on patients most likely to deteriorate and require enhanced ventilatory support.

A limitation of our analysis is that it lacked internal or external validation. Initial models often over-fit data and prove less reliable when applied to out-of-population datasets. The model is nonetheless likely to remain useful, even if somewhat less predictive in other COVID populations. Practical implementation of our model will require software built-into the Circadia system or medical record systems. Our analysis was restricted to patients with proven SARS-CoV-2 infections. Results will likely differ with other respiratory viral infections, and of course other respiratory diseases. Nonetheless, the general concept that continuous respiratory rate monitoring is prognostic of ventilatory decompensation over a range of conditions is probably valid—although model specifics and coefficients will presumably differ for each.

In summary, our pilot observational study suggests that continuous respiratory monitoring and derived features are associated with the need for care escalation 24 in advance. Out-of-sample validation remains necessary. But in the meantime, our results suggest that continuous respiratory monitoring is a valuable increment over intermittent monitoring.

## Data availability statement

The raw data supporting the conclusions of this article will be made available by the authors, without undue reservation.

## Ethics statement

The study was conducted with approval from the Hospital Clinic of Barcelona IRB (Ethics Committee’s approval/ID: HCB 2020/0666). Written informed consent was obtained from participating patients.

## Author contributions

ER helped to conceive and design the study, collect, analyze and interpret the data, and write and critically revise the manuscript. ML-B helped to collect and interpret the data, and write and critically revise the manuscript. JS, ER, TR, AR, AL, AS, JB, PC, and MA helped to collect the data, and write and critically revise the manuscript. SK helped to analyze and interpret the data. TL and DS helped to conceive and design the study, analyze and interpret the data, and write and critically revise the manuscript. OA helped to analyze the data. EJM helped to conceive and design the study, analyze and interpret the data, and write and critically revise the manuscript. All authors contributed to the article and approved the submitted version.
