# Supplementary material for: Early detection of deterioration in COVID-19 patients by continuous ward respiratory rate monitoring: a pilot prospective cohort study
Source: Front Med (Lausanne). 2023 Oct 31;10:1243050. doi: 10.3389/fmed.2023.1243050 (PMC10645134; doi:10.3389/fmed.2023.1243050)
Supplement: Supplementary file 1 [file Data_Sheet_1.PDF]

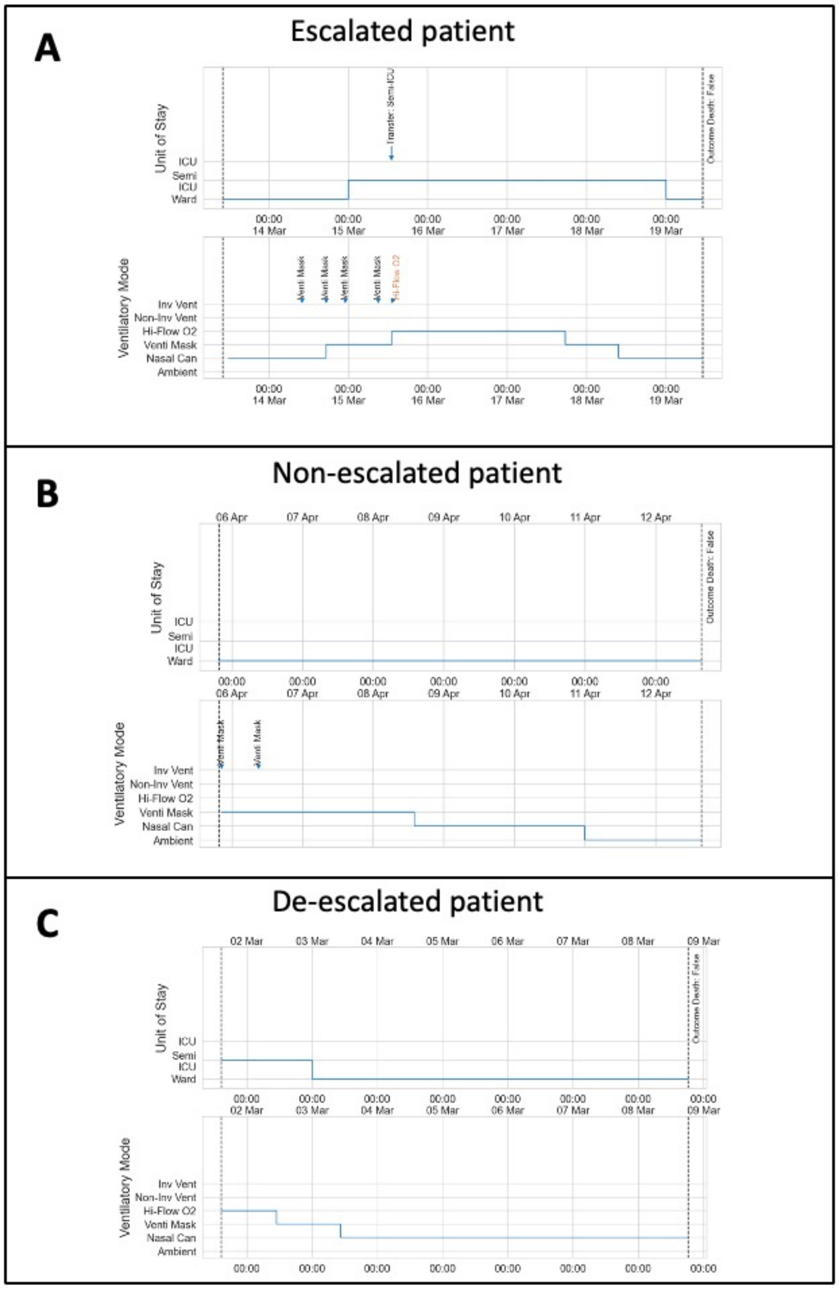

Supplemental figure 1. A panel, shows an example of an escalating patient who at the study inclusion time, while being in the ward needed ventilatory support with venti mask, but later on he was moved to a semi-ICU ward to start ventilatory support with high-flow nasal cannula ventilation; **B panel,** is an example of a non-escalating patient. The patient joined the study when he was in the ward on ventimask and he did not need any further support; and **C panel** shows a patient who joined the study when he was on a Semi-ICU unit on Hi-flow oxygen, then he moved to a ward with venti mask + and no additional ventilatory support.

Supplement

Supplemental table 1. Respiratory rate features

Feature name	Description
**Mean, 30 minutes**	Mean across 30 minutes of raw RR data
**SD, 30 minutes**	Standard deviation across 30 minutes of raw RR data
**Skew, 30 minutes**	Skewness across 30 minutes of raw RR data
**Kurtosis, 30 minutes**	Kurtosis across 30 minutes of raw RR data
**Mean, 1 day**	Mean across 24 hours of down-sampled RR data
**SD, 1 day**	Standard deviation across 24 hours of down-sampled RR data
**Skew, 1 day**	Skewness across 24 hours of down-sampled RR data
**Kurtosis, 1 day**	Kurtosis across 24 hours of down-sampled RR data
**Trend, 1 day**	Slope, obtained using linear fit, in bpm per day, across 24 hours of down-sampled RR data
**Mean, 3 days**	Mean across 72 hours of down-sampled RR data
**SD, 3 days**	Standard deviation across 72 hours of down-sampled RR data
**Skew, 3 days**	Skewness across 72 hours of down-sampled RR data
**Kurtosis, 3 days**	Kurtosis across 72 hours of down-sampled RR
**Trend, 3 days**	Slope, obtained using linear fit, in bpm/day, across 72 hours of down-sampled RR data

RR, respiratory rate; SD, standard deviation; bpm, breaths per minute. All units are in bpm, except for Trend (bpm/day). Down-sampled RR data denotes 30-minute RR averages, corresponding to the ‘Mean, 30 minutes’ feature.

Supplemental Table 2. Matching of escalated patients with non-escalation patients using Euclidean distance.

PATIENT	E1	E2	E3	E4	E5	E6	E7	E8	E9	E10	E11	E12	E13
NE1	2.32	2.22	2.38	2.32	2.21	2.31	2.23	1.99	2.24	2.51	2.84	2.02	2.10
NE2	2.41	1.75	2.08	1.26	2.63	1.76	1.84	2.45	2.24	2.19	2.82	2.10	2.64
NE3	2.48	1.10	2.60	2.04	2.61	1.01	1.80	2.47	2.49	2.79	2.79	2.13	2.48
NE4	2.24	1.16	2.19	2.01	2.14	1.38	1.92	2.43	2.67	2.53	2.67	1.66	2.32
NE5	2.05	2.14	2.06	2.34	1.92	2.10	2.10	2.64	2.76	2.97	2.46	1.97	1.90
NE6	2.46	2.56	1.99	2.46	2.85	2.70	2.63	2.39	2.03	2.55	2.68	2.49	2.63
NE7	2.57	1.62	2.08	1.29	2.39	1.72	1.58	2.75	2.57	2.03	2.97	1.93	2.58
NE8	2.32	1.89	1.68	1.36	2.16	1.97	1.84	2.62	2.53	2.14	2.88	1.48	2.54
NE9	2.38	1.92	2.16	2.07	1.60	2.02	1.88	2.59	2.90	2.63	2.89	1.71	1.85
NE10	2.38	1.89	2.31	2.15	1.65	2.00	1.90	2.49	2.69	2.79	2.76	1.78	1.67
NE11	2.62	1.93	2.27	1.73	2.64	1.99	1.89	2.76	1.98	2.43	2.99	2.30	2.51
NE12	2.28	1.88	2.19	2.03	1.59	1.92	1.80	2.57	2.76	2.64	2.80	1.54	1.92
NE13	2.50	2.15	1.98	1.93	2.44	2.30	2.15	2.20	2.00	2.02	2.93	2.05	2.42
NE14	2.59	2.32	2.59	2.55	2.26	2.50	2.44	2.03	2.19	2.73	2.72	2.32	1.92
NE15	2.60	0.79	2.53	1.92	2.34	1.06	1.62	2.82	3.03	2.38	3.05	1.91	2.66
NE16	2.51	2.19	2.39	2.37	1.97	2.34	2.28	2.66	2.92	2.79	2.58	2.11	1.39
NE17	2.49	1.52	1.96	0.88	2.36	1.68	1.53	2.67	2.57	1.93	2.96	1.82	2.64
NE18	2.63	2.67	2.59	2.78	2.57	2.64	2.72	2.72	3.21	3.29	1.88	2.48	2.17
NE19	2.35	2.15	2.44	2.48	2.09	2.22	2.22	2.30	2.33	3.00	2.51	2.16	1.41
NE20	2.55	2.12	1.92	1.72	2.37	2.34	2.18	2.37	2.38	1.68	3.09	1.92	2.74
NE21	2.10	2.59	2.33	2.61	2.35	2.55	2.54	3.22	3.51	2.99	3.08	2.25	2.72
NE22	2.37	2.09	2.47	2.42	1.97	2.09	2.17	2.33	2.34	3.00	2.46	2.06	1.40
NE23	2.68	1.64	2.60	1.98	1.84	1.70	1.60	2.83	2.99	2.59	3.02	2.04	2.06
NE24	2.71	2.79	1.68	2.51	3.05	2.89	2.76	2.83	2.70	2.76	2.77	2.67	2.67
NE25	2.43	1.94	2.21	2.01	1.68	2.04	1.96	2.64	3.00	2.48	2.89	1.61	2.15
NE26	1.86	2.41	2.21	2.57	2.25	2.57	2.45	2.92	3.19	2.94	2.90	2.26	2.45

This table shows the 13 escalated patient in columns and the 26 non-escalated patients in the rows. Each escalated patient was matched with the 3 non-escalated patients with the lowest Euclidean distance (brown color). Non-escalated patient could be matched with more than 1 escalated patient. Specifically, 2 non-escalation subjects were matched 3 times to an escalated subject, 9 non-escalated subjects matched twice, and the remaining 15 were matched only once. E, escalated patients; NE, Non-escalated patients.
